# Enhancement of MCF Rubber Utilizing Electric and Magnetic Fields, and Clarification of Electrolytic Polymerization

**DOI:** 10.3390/s17040767

**Published:** 2017-04-04

**Authors:** Kunio Shimada

**Affiliations:** Faculty of Symbiotic Systems Sciences, Fukushima University, 1 Kanayagawa, Fukushima 960-1296, Japan; shimadakun@sss.fukushima-u.ac.jp; Tel.: +81-24-548-5214

**Keywords:** sensor, electrolytic polymerization, magnetic field, magnetic cluster, natural rubber, magnetic compound fluid (MCF), magnetic fluid, isoprene, filler, electrical resistivity, oxidation-reduction reaction

## Abstract

Many sensors require mechanical durability to resist immense or impulsive pressure and large elasticity, so that they can be installed in or assimilated into the outer layer of artificial skin on robots. Given these demanding requirements, we adopted natural rubber (NR-latex) and developed a new method (NM) for curing NR-latex by the application of a magnetic field under electrolytic polymerization. The aim of the present work is to clarify the new manufacturing process for NR-latex embedded with magnetic compound fluid (MCF) as a conductive filler, and the contribution of the optimization of the new process for sensor. We first clarify the effect of the magnetic field on the enhancement of the NR-latex MCF rubber created by the alignment of magnetic clusters of MCF. Next, SEM, XRD, Raman spectroscopy, and XPS are used for morphological and microscopic observation of the electrolytically polymerized MCF rubber, and a chemical approach measuring pH and ORP of the MCF rubber liquid was used to investigate the process of electrolytic polymerization with a physical mode. We elucidate why the MCF rubber produced by the NM is enhanced with high sensitivity and long-term stability. This process of producing MCF rubber by the NM is closely related to the development of a highly sensitive sensor.

## 1. Introduction

Sensors are currently used in many fields to detect force, temperature, pressure, etc. However, in robotics, sensors must have the mechanical durability to withstand immense or impulsive pressure, as well as large elasticity or extensibility. Certain sensors should be suitable for installation as an outer layer on robots, or for assimilation into an artificial skin. These characteristics are not, of course, limited to the sensors in robotics; sensors with mechanical durability are also required in other engineering fields. Regarding the concept of a skin sensor, there has been only a few general investigations [1]. The paradigm is that the sensor should be based on the effective control or management of human-machine systems. One new natural-contact biosensor has been designed as an interface between humans and machines for nonintrusive and real-time sensing. Although this sensor utilizes microelectromechanical systems (MEMS) or nanoelectromechanical systems (NEMS) to enable miniature sensors, flexibility remains a fundamental problem. 

Therefore, as described above, there is a need for a suitable rubber material with the durability to withstand impulsive, immense or repeated force while maintaining large elasticity and extensibility [2]. 

Researchers have examined many materials in the search for a sensor made of rubber: silicon oil rubber [3,4,5,6,7,8], chloroprene [9], styrene-butadiene [10], acrylonitrile butadiene [11], nitrile butadiene rubber [12], and natural rubber (NR-latex) [13,14,15,16]. Shimada proposed a new sensor made of silicon-oil rubber that uses magnetic compound fluid (MCF) as filler (called MCF rubber) [17]. MCF was devised in 2001 as a colloidal fluid containing Fe_3_O_4_ sphere particles on the order of 10 nm, coated by oleic acid and other metal particles such as Ni, Fe, and Cu, on the order of 1 μm. These particles are dispersed in a solvent such as water, kerosene, or silicone oil to compound a magnetic fluid (MF), which is an ordinary intelligent fluid responsive to the magnetic field with Fe_3_O_4_ particles. When a magnetic field is applied, the Fe_3_O_4_ particles play a bonding role among the metal particles, causing numerous metal and Fe_3_O_4_ particles to aggregate [18]. Due to these magnetic clusters, MCF is useful for many engineering applications [19]. The mechanism of electric conductivity of silicon-oil type MCF rubber is explained mainly by the percolation theory [20] and tunnel theory [21]. Percolation threshold is significant factor in the former. Therefore, in the case of a small mass concentration of MCF rubber, it is possible that electric conductivity is demonstrated by this theory. On the other hand, for a large aggregation of particles with large aspect ratio, the latter theory can better explain the electric conductivity of MCF rubber. Electrons jump between metal particles such as Fe_3_O_4_ and Ni through the barrier of non-conductive materials such as silicon-oil rubber and oleic acid coated around Fe_3_O_4_.

Silicon-oil type MCF rubber has a large electrical resistivity, however, which does not fulfill the needs of a skin sensor. In order to obtain sensitivity at a shearing motion, such as touching a body and scrubbing, a skin sensor requires high sensitivity, as shown by a previous investigation. When two electrodes are set between skin rubber and supplied with voltage, the electric current flowing inside the rubber can be measured. The electrical conductivity changed by the force applied on the skin rubber is related to the degree of sensitivity. 

In order to achieve the requirement of high sensitivity, we can enhance the sensitivity of MCF rubber using NR-latex. NR-latex type MCF rubber is made by solidification under a constant temperature (the drying method (DM)) and the application of a magnetic field. The DM has also been used to produce ordinary, commercial, pressure-sensitive electrically conductive rubber (PSECR). The application of the magnetic field causes the Fe_3_O_4_ and metal particles to aggregate, and magnetic clusters are created in NR-latex type as well as silicon-oil type MCF rubber. In general, water involved in NR-latex plays an important role in electric conductivity, which is based on ionic conduction. Therefore, owing to the filler and the water role, electric conductivity is superior to that of PSECR. Furthermore, as shown in a previous investigation, NR-latex type MCF rubber has been evaluated the following technical cases as a physical value: surface roughness of a hard plate surface with coarse or smooth roughness in the range of *R_a_* = 0.19–20.86 µm, which is related to finishing in precision machinery industry; softness of a soft surface of texture of clothes, paper, gel, etc.; geometric shape of concave and convex surface less than 5.7 mm in height, which is related to the evaluation of a biological surface such as a fish, insect, etc. 

NR-latex type MCF rubber made by the DM, however, has a problem in that deterioration occurs in a short period of about a week. In addition, it has electrical resistivity on the order of 1 Ω·m [22], which does not provide high enough sensitivity to fulfill the requirements for a skin sensor. Shimada has proposed a new method (NM), which involves solidifying rubber using both magnetic and electric fields. The rubber liquid is poured between electrodes, and a magnetic field is applied. NR-latex without MCF is created as a thin film, and its thickness does not increase even with an extended application of the electric field. With NR-latex MCF rubber, however, since the magnetic field is applied along the same direction as the electric field line, the thickness increases substantially due to the magnetic clusters. When the electric field or magnetic field is used alone, the electrical property is less sensitive than with both, and the mechanical property is less extensible. These characteristics have been shown in the case of a narrow electrode gap of 1 mm in previous studies [22]. It was also clarified that NR-latex MCF rubber made with NM maintains a smaller electrical resistivity on the order of 10^−2^ − 10^−3^ Ω·m from its creation to a period of more than a year. The NM is a noteworthy and effective production method for avoiding deterioration due to aging.

However, how enhancement of MCF rubber thickness is affected by various strengths of the electric and magnetic fields has not yet been clarified. While the effect of the magnetic field on electrolytic polymerization is significant, parameters including the magnetic field distribution and magnitude have not yet been elucidated. In particular, the phenomenon of how MCF rubber is enhanced by both fields at a large electrode gap remains unknown. Investigating the enhancement of MCF rubber at a large electrode gap is related to clarifying the configuration of the enhancement. Therefore, in the present paper, we first investigate the effect of the magnetic field on the metamorphosis of MCF rubber by electrolytic polymerization.

On the other hand, magnetic clusters in the filler play a very significant role in the solidification of MCF rubber with the NM. Controlling the applied magnetic field allows the magnetic clusters to grow freely in the filler. In terms of whether the filler is magnetic or conductive, there have been many studies on the use of polymers such as polymethyl methacrylate (PMMA). Carbon nanotubes is another well-known filler [23]. The properties of a sensor can be controlled by the alignment of the carbon nanotube with a magnetic field. The idea and concept of embedding fillers of carbon nanotubes in a polymer is similar to that of the MCF rubber. Only the material of the solvent and substrate is different. There has been research that looked at carbon nanotube in NR-latex [24,25,26,27,28]. Most of these studies, however, did not consider the alignment of the filler with the aid of a magnetic field. As for NR-latex with magnetic particles other than carbon nanotube, there have also been some studies [29,30,31,32,33]. A few dealt with the alignment of the filler with the help of a magnetic field. However, investigation of filler alignment with the help of a magnetic field under electrolytic polymerization such as the NM has not been clarified. Therefore, the present paper contributes to the optimization of a new manufacturing process for an NR-latex embedded with MCF as a conductive filler by adding electric and magnetic fields. 

From mentioned above, the mechanisms behind the enhancement, long-term stability and high sensitivity of electric conductivity have not been elucidated, nor the process of electrolytic polymerization of NR-latex type MCF rubber been clarified. In our previous study [22], we could only guess at the physical model. Clarification of this is related to the development of high-sensitivity sensors, or skin sensors if you will. Therefore, the second aim of the present study is to investigate the physical model, and the processes of enhancement of electrolytically polymerized MCF rubber by morphological and microscopic examination, including observations by SEM, XRD, Raman spectroscopy and XPS, as well as by chemical analysis including measurement of pH and ORP of the MCF rubber liquid.

## 2. Effect of Magnetic Field on Metamorphosis of MCF Rubber

**Figure 1:**
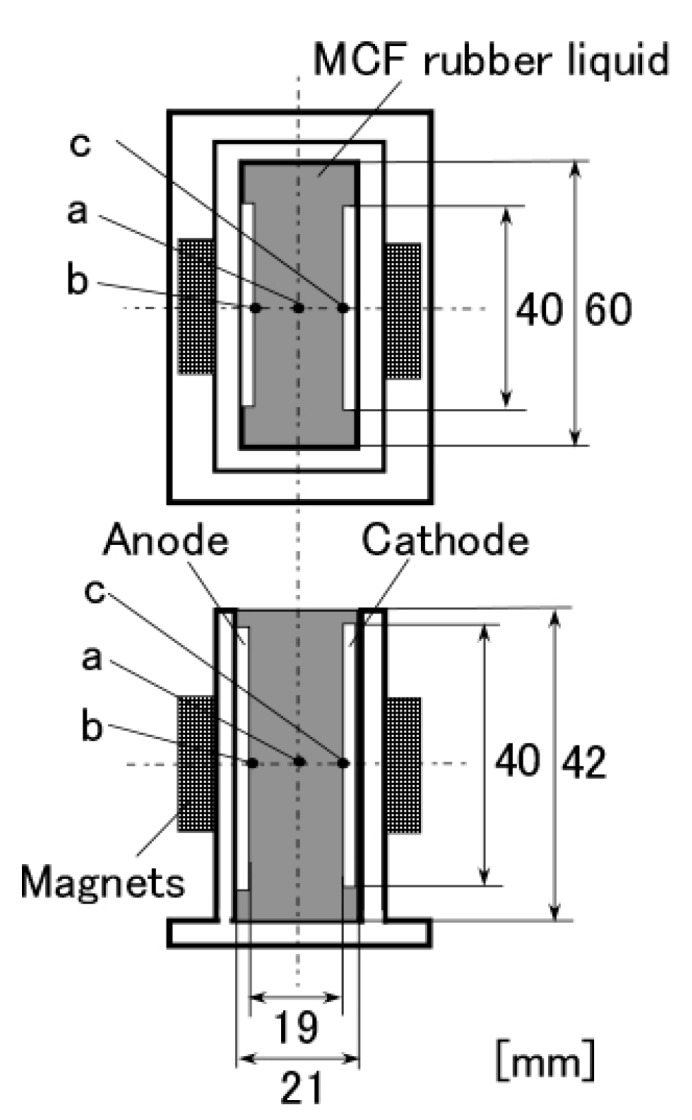
Schematic diagram of experimental apparatus used to investigate the effect of magnetic field distribution and magnitude on the solidification of MCF rubber liquid by the NM.

**Figure 2:**
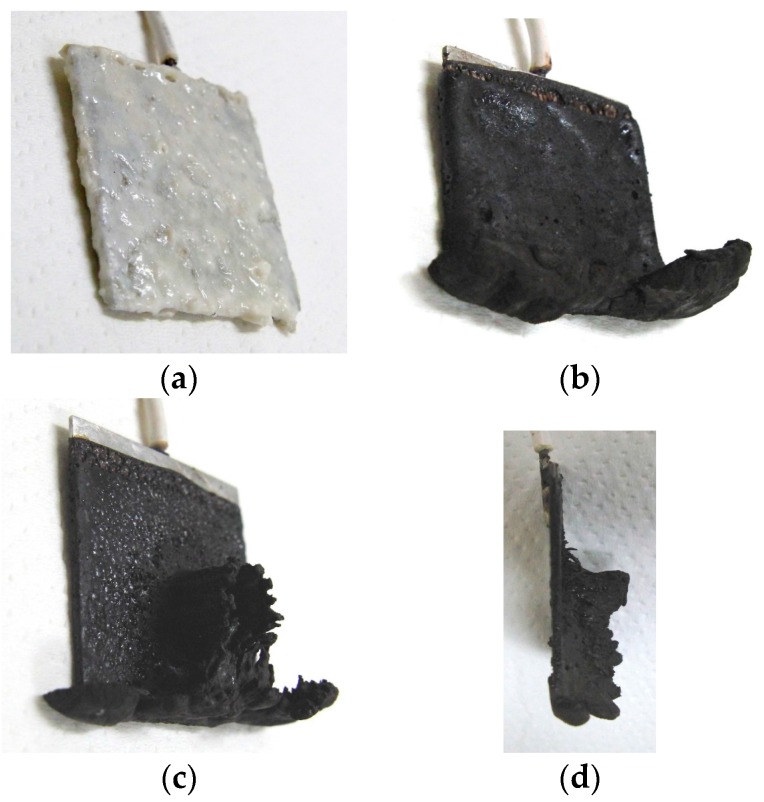
Photographs of electrolytically polymerized rubber: (**a**) NR-latex without application of a permanent magnet; (**b**) MCF rubber without application of a permanent magnet; (**c**) MCF rubber with application of a permanent magnet; (**d**) image from viewing transversely to the electrode of (**c**). NR-latex or MCF rubber solidified and attached to an anode electrode after electrolytic polymerization under the conditions in which the electrodes are immersed transversely in a container (see Figure 1).

It is clear from the results of previous studies [22] that the thickness of MCF rubber solidified by electric and magnetic fields depends on the magnetic field. However, the effect of magnetic field distribution and magnitude on the enhancement of solidified MCF rubber is not clear. In our previous study [22], we applied permanent magnets to the inside of plates to form the outer surface of each plate. The liquid in the MCF rubber consisted of 12-g Ni powder, with particles on the order of µm and pimples on the surface (No. 123, Yamaishi Co., Ltd., Noda, Japan), 3-g water-based MF with 50 wt % Fe_3_O_4_ (M-300, Sigma Hi-Chemical Co., Ltd., Tsutsujigasaki, Japan), 16-g NR-latex (Rejitex Co., Ltd., Atsugi, Japan), and 31-g water, poured into a container as shown in Figure 1. The mass concentration of MCF rubber liquid was 21.7 wt % of Ni and Fe_3_O_4_ particles. Electrodes were immersed in the liquid. Permanent rectangular magnets, 10 mm × 15 mm in size, and 5-mm thick, were applied from outside the container. An electric field held constant at 6 V voltage and 2.7 A electric current was supplied between the plates for 30 min. The plates were held apart at a constant thickness with a spacer to create an electrodes gap of 19 mm, and a 40 mm × 40 mm square made of stainless steel. In the case of NR-latex only, and the application of an electric field without a magnetic field, the NR-latex created an anode plate as a thin film, as shown in Figure 2a. In contrast, compounding MCF into the NR-latex with an electric but not magnetic field increased the thickness of the MCF rubber to 2 mm at the electrode surface, double the NR-latex alone, as shown in Figure 2b. The enhancement of the MCF solidified rubber at the lower side was due to the sedimentation of the MCF at the bottom of the container. This enhancement did not depend on the magnetic field, as it was not applied. This phenomenon is investigated later in the subsequent figure from these figures. 

Meanwhile, by compounding MCF into the NR-latex and applying both an electric and magnetic field (Magnet 2 in Table 1), the thickness of the MCF rubber grew significantly along the magnetic field line, as shown in Figure 2c. NR-latex without MCF generally forms as a thin film, and its thickness does not increase even with a longer application of the electric field. Therefore, the application of the magnetic field is necessary to create MCF magnetic clusters. Since the magnetic field is applied along the same direction as the electric field line, as may be seen in the figure, the thickness of the MCF rubber grows substantially. The NM is related to so-called electric polymerization. The curing of NR-latex is promoted by the alignment of magnetic clusters in the filler due to the application of a magnetic field under electrolytic polymerization.

**Figure 3:**
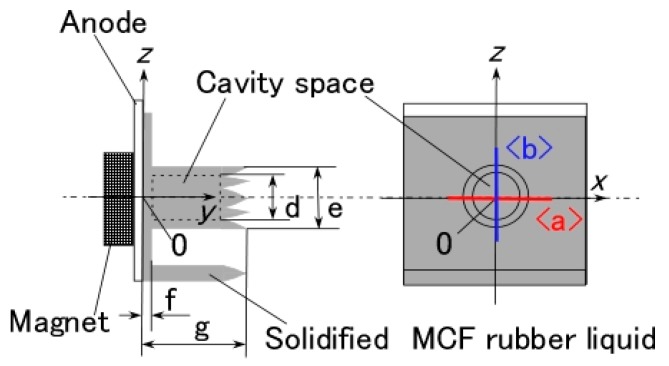
Schematic diagram of MCF rubber liquid solidified using the NM. The position for measuring magnetic field distributions shown in Figure 5 is also delineated.

**Figure 4:**
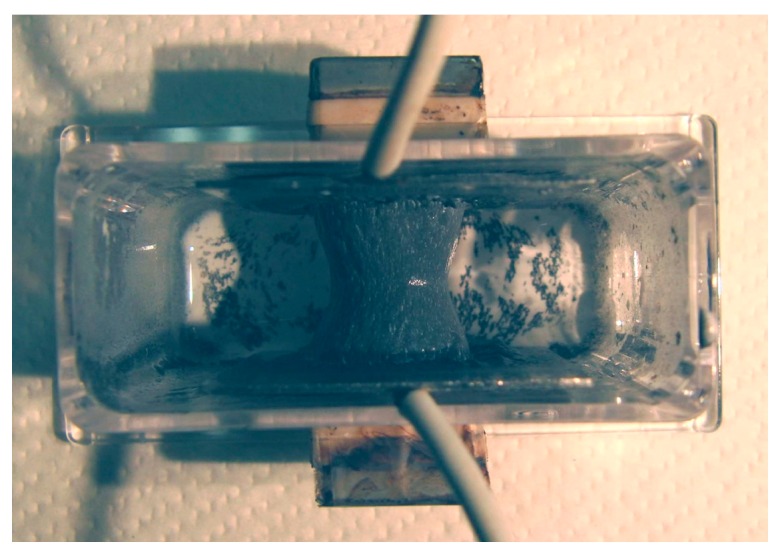
Photograph of MCF rubber liquid attracted to the electrodes before the application of electric field.

Magnet 2 used to create the NR-latex shown in Figure 2c had a magnetic field strength of 188 mT, based on previous studies [22]; the one at the surface 107.5 mT in the case of a single magnet. The size of the solidified MCF rubber in Figure 2c corresponded *d* = 8 mm (diameter), *e* = 13 mm (diameter), *f* = 1.5 mm, *g* = 13 mm in Figure 3. Before application of the electric field, MCF rubber liquid was attracted to the electrodes attached to Magnet 2 on the outside of the container, as shown in Figure 4. If both a magnet with the magnetic field strength of 130 mT used in previous studies [22] with a single surface magnet of 84.7 mT (Magnet 1) were used, the same solidified MCF rubber would be created. However, if magnetic field strengths of 312 mT and 177.7 mT (Magnet 3), or 490 mT were used, [22] together with a surface magnet of 191.3 mT (Magnet 4), solidified MCF rubber would not form. The magnetic field strength of Magnets 1–4 are listed in Table 1. We found that MCF rubber would not solidify when too large of a magnetic field strength was applied. When magnetic field strength was less than about 50 mT, MCF rubber liquid solidified. However, this was in the case of 21.7 wt % particles. As a result, the solidification of MCF rubber liquid was considered to depend on magnetic field strength. 

**Figure 5:**
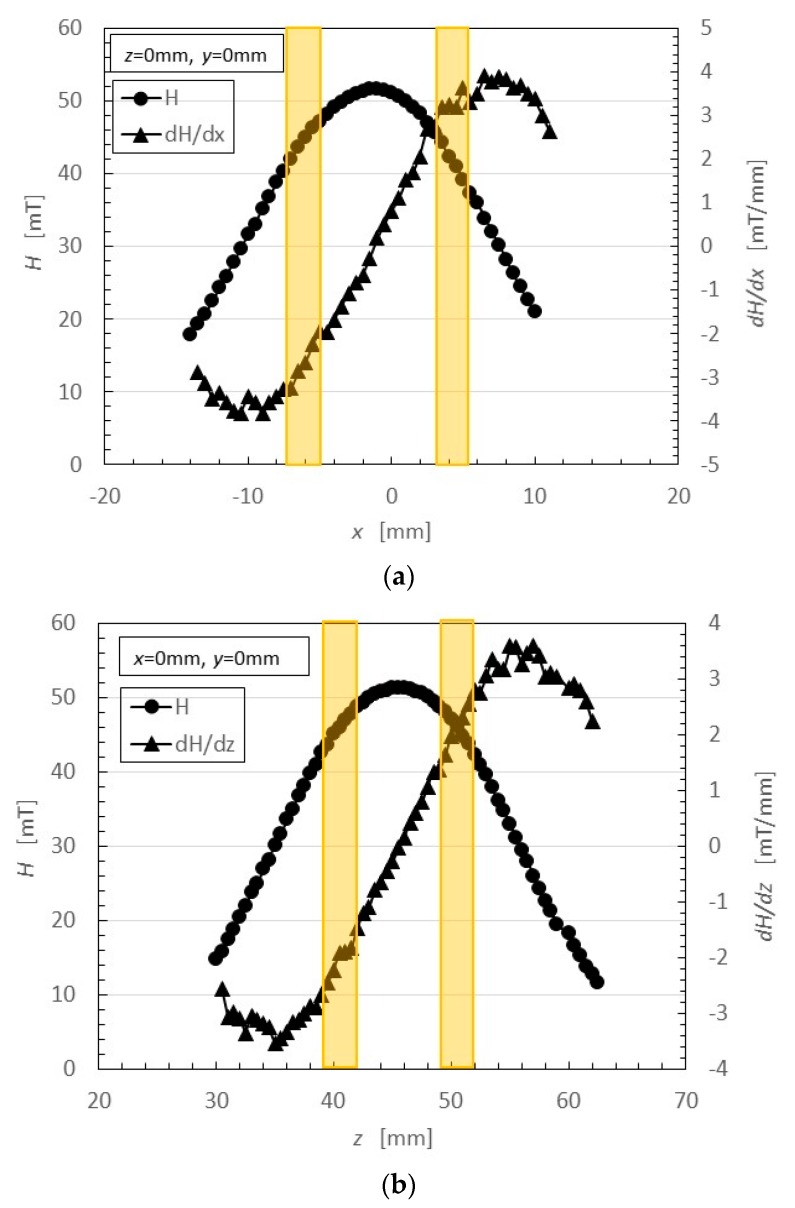
Magnetic field distribution of Magnet 2 (**a**,**b**) corresponding to the location labeled <a> and <b> in Figure 3.

In the case of Magnet 2, the magnetic field distribution at position <a> and <b> in Figure 3 was measured; the results are shown in Figure 5. The yellow bands in Figure 5 correspond to the positions at which the solidified MCF rubber increased as in Figure 2c. These results suggest that the MCF rubber grows at the position between the maximum magnetic field strength and the maximum magnetic field gradient. It is considered that this is due to the aggregation of the Ni and Fe_3_O_4_ particles by the force of the magnetic field strength and field gradient. The permanent magnet used had a circular maximum magnetic field and field gradient. Therefore, the solidified MCF rubber was cylindrical in shape (Figure 3).

On the other hand, the enhancement of the solidified MCF rubber can be perceived at the lower side of the solidified MCF rubber shown in Figure 2c, as well as in the case without application of magnetic field shown in Figure 2b, due to the sedimentation of the particles in the MCF rubber. Therefore, large mass concentration is another factor that enhances the solidification of MCF rubber. 

**Figure 6:**
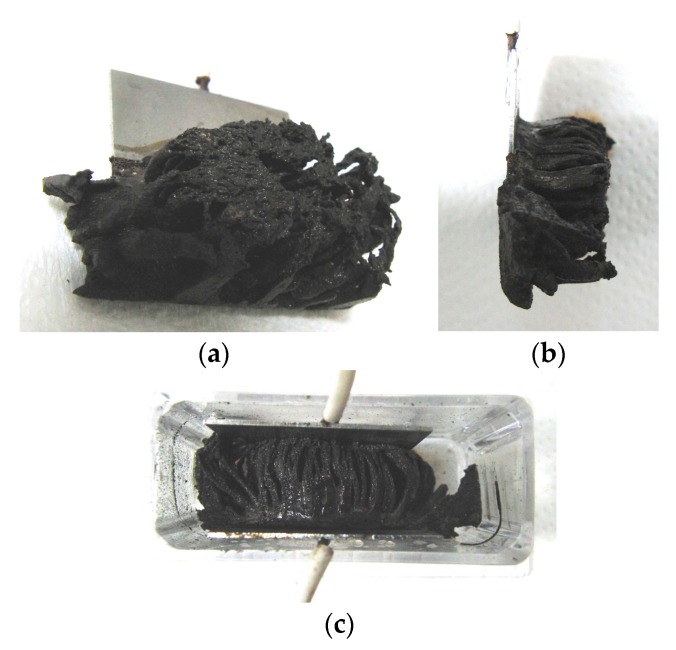
Photographs of solidified MCF rubber with large mass concentration: (**a**,**b**) attracted on the anode; (**c**) between the electrodes.

Figure 6a shows MCF rubber solidified by the NM; this rubber was attracted on the anode of Magnet 2 in an MCF rubber liquid with 50 wt % larger mass concentrations of particles, consisting of 12-g Ni powder, 3-g MF (M-300) and 12-g NR-latex. In contrast, particles in the MCF rubber liquid in Figure 2 were 21.7 wt %. The solidified MCF rubber in Figure 6 was larger than that in Figure 2c, reaching from anode to cathode with *g* = 19 mm. Thus, the higher the mass concentration, the larger the solidified MCF rubber.

The solidified MCF rubber in Figure 6a,b, which shows the rubber between the electrodes, has the same wire-like shape as that shown in Figure 2c. Where the magnetic field is applied, needle-shaped magnetic clusters are created. Meanwhile, in areas outside of the magnetic field, such as the lower side of the solidified MCF rubber shown in Figure 1b, no needle-shaped magnetic clusters form, and the particles aggregate into a dense state. Note that if the electrode gap is small, like the 1-mm gap used in previous studies [22], the solidified MCF rubber reaches from anode to cathode and becomes a solid, 1-mm thick plate. In that case, the needle-like magnetic clusters are confined between the electrode plates, as shown in our previous study [22].

**Figure 7:**
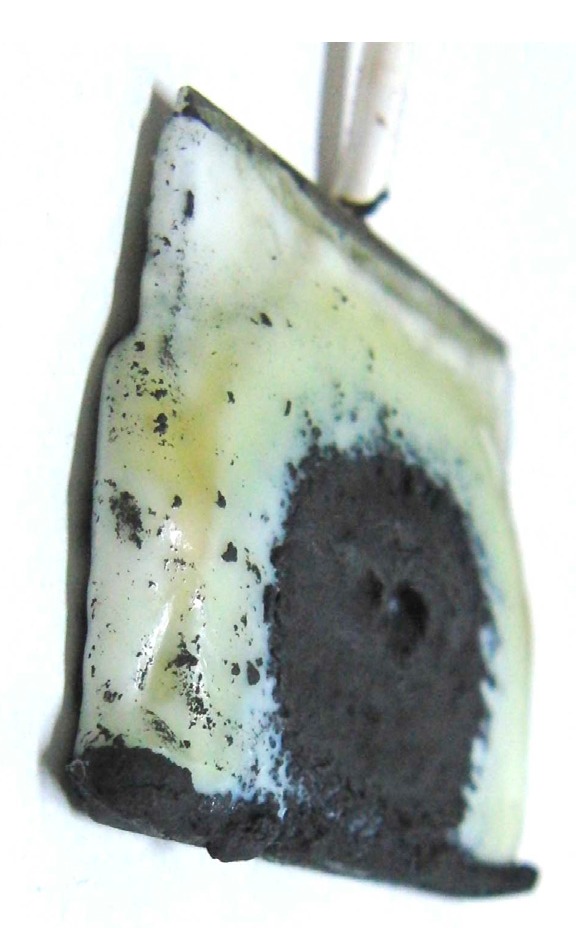
Photograph of solidified MCF rubber without MF.

When the MCF rubber liquid does not include MF, the phenomena of the electrolytically polymerized configuration is different, as shown in Figure 7, where the MCF rubber liquid consisted of 12-g Ni powder, 16-g NR-latex and 34-g water. Magnet 2 was used here. The black area is Ni and the white or pale yellow is NR-latex. Even if the Ni particles were aggregated by the application of the magnetic field, the solidified MCF rubber would not grow. This suggests that MF is the important factor in the enhancement of the solidification of MCF rubber.

Thus, we have demonstrated that MCF rubber liquid with 21.7 wt % can be solidified at less than about 50-mT magnetic field strength and enhanced at the position between the maximum magnetic field strength and maximum magnetic field gradient. The rubber grows substantially with a higher mass concentration of particles in the MCF rubber liquid. The MF in the MCF rubber liquid is a very important factor in the enhancement of solidified MCF rubber with a wire-like shape.

## 3. Mechanisms and Process of Electrolytically Polymerized MCF Rubber

**Figure 8:**
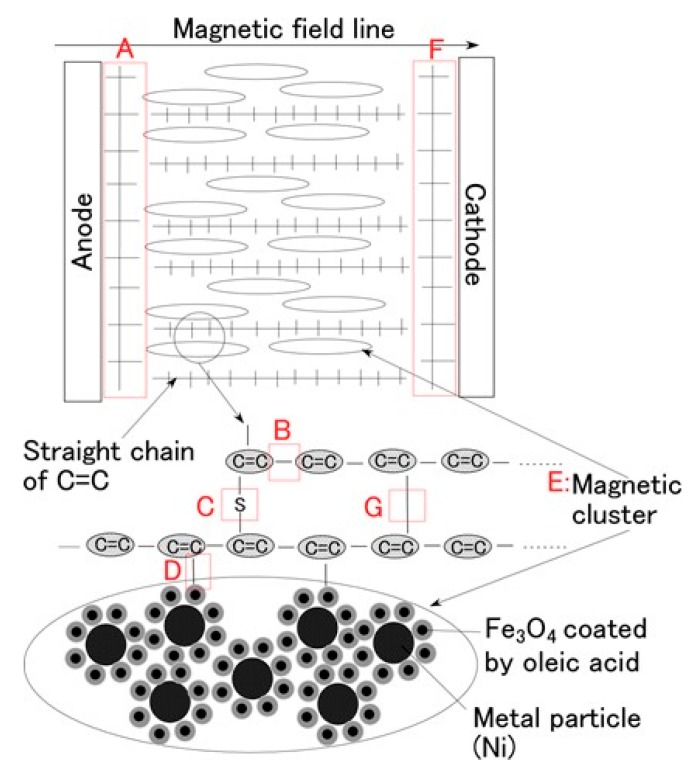
Physical model of electrolytic polymerization of the MCF rubber by the NM [22]. Note that G in the figure was added in the present study.

In this section, we first describe the electrolytic polymerization model of MCF rubber, because all the following investigations are based on this model. Figure 8 shows the physical model of the solidification of MCF rubber by electrolytic polymerization proposed in our previous study [22]. Using this model can help to clarify **t**he mechanisms behind the MCF rubber enhancement, long-term stability and high sensitivity. With the application of the magnetic field, the magnetic clusters align along the lines of the magnetic field, which runs in a transverse direction to the two electrodes, as indicated by E in the figure. The C=C are arrayed in the same direction as the magnetic clusters. As a result, solidification of the MCF rubber is guessed to expand in the direction of the applied magnetic field. When a magnetic field is not applied, the C=C bonds simply undergo solidification, and the direction of the polymerization is random. As a result, the thickness of the solidified MCF rubber and the solidification effect are very small. At the cathode, the surface of the MCF rubber becomes rugged, as shown in [22], due to the tip of the growing magnetic clusters. Cationic polymerization occurs at the anode, and anionic polymerization at the cathode (A and F in Figure 8). With the application of electricity, each polyisoprene of a particle in the NR-latex is guessed to be mutually polymerized to bind between the ends of C=C bonds in a coordinated way, and many C=C bonds be formed in a long chain, as indicated by B in Figure 8. At the same time, the C=C bonds are vulcanized by radical polymerization, as shown by G in Figure 8. If the NR-latex includes sulfur S, the C=C bonds are vulcanized by radical polymerization, as shown by C in Figure 8. In addition, from the results of the last Section 2, where we showed that enhancement of the solidified needle-shaped MCF rubber depends on the MF in the MCF rubber liquid, it can be guessed that radical polymerization occurred as a result of the carboxyl group COOH of the oleic acid coating Fe_3_O_4_, bonding C=C, and Fe_3_O_4_, as shown by D in Figure 8.

**Figure 9:**
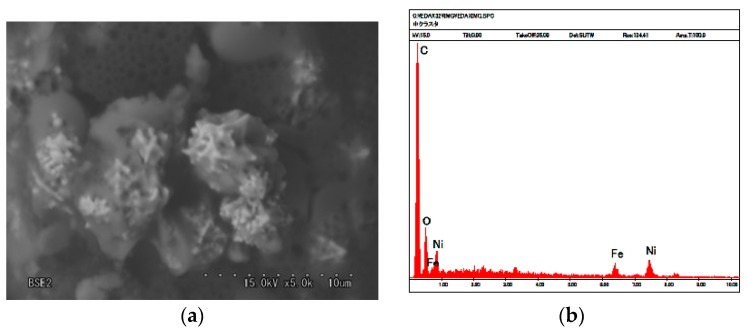
(**a**) SEM photograph (5000× magnification of 5000) of microspores of magnetic clusters inside solidified MCF rubber; (**b**) XRD mapping of (**a**).

As shown in our previous study [22], the magnetic clusters that align along the magnetic field form needle-like structures. Figure 9a is an SEM image of part of a magnified magnetic cluster involved in the electrolytically polymerized solid MCF rubber of the image shown in our previous study [22]. The MCF rubber liquid consisted of 12-g Ni, 3-g MF (M-300) and 12-g NR-latex. MCF rubber was electrolytically polymerized by a 30-min application of 6 V and 2.7 A, a 1-mm space between electrodes, and Magnet 2. The molecules of the NR-latex coiled around the magnetic clusters. Figure 9b shows the mapping by X-ray diffraction (XRD) of Figure 9a. The electrolytic polymerized MCF rubber presents a complicated configuration of intertwined NR-latex, Fe_3_O_4_ and Ni, indicating that these ingredients of NR-latex do not exist independently.

**Figure 10:**
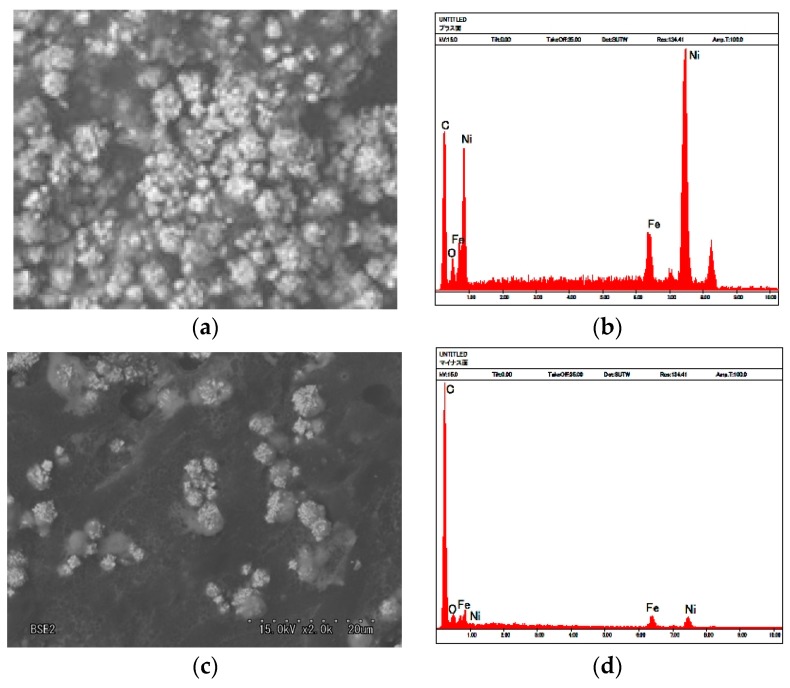
Photographs of (**a**) anode-side and (**c**) cathode-side surfaces of solidified MCF rubber by SEM (2000× magnification); (**b**) XRD mapping on (**a**); (**d**) XRD mapping on (**c**).

Figure 10a,b present SEM and XRD images of the anode side surface, corresponding to A in Figure 8, and Figure 10c,d are those of the cathode side, corresponding to F in Figure 8. The MCF rubber sample is that shown in Figure 9. Figure 10b shows that on the anode-side surface, the quantity of Ni and Fe_3_O_4_ particles involved in the NR-latex is larger than that of C. In contrast, Figure 10d shows that on the cathode-side surface, the quantity of C is larger than that of Ni and Fe_3_O_4_ particles. The electrolytic polymerization of NR-latex is thus thought to be different at the anode and cathode sides of the MCF rubber. This is the typical distinction.

We next investigate the MCF rubber by chemical analysis. Regarding the electrolytic polymerization of NR-latex, there have been only a few invaluable reports [34,35]. In contrast, there have been several investigations of NR-latex as a composite without chemical polymerization [13,14,15,16]. The former [34,35] are useful because of their description of the results of FT-IR spectra, etc. However, the structure of the electrolytic polymerization of NR-latex has not been clarified. To select the optimum analytical instrument, it is important to consider that MCF rubber involves magnetic material, 0.1-mm thick, and created instantaneously by the application of an electric field. We thus selected Raman spectroscopy and XPS (X-ray photoelectron spectroscopy) as effective measures for MCF rubber. 

**Figure 11:**
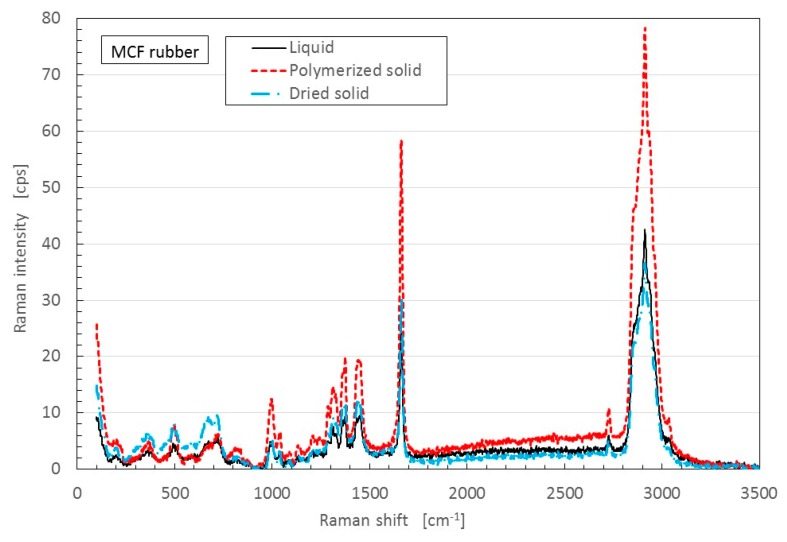
Raman spectroscopy spectra of the electrolytically polymerized MCF rubber of anode-side surface indicated as “Polymerized solid”: MCF rubber liquid before the application of electric and magnetic fields as “Liquid”; MCF rubber of anode-side surface solidified by the DM with a magnetic field as “Dried solid”.

**Figure 12:**
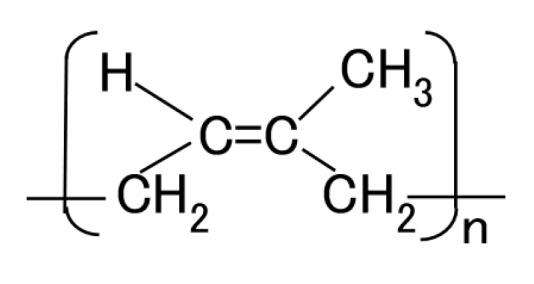
Scheme of polyisoprene structure of NR-latex.

Raman spectroscopy spectra were taken from the anode-side surface of the MCF rubber electrolytically polymerized under the conditions described in the last Section 2; the MCF used consisted of 12-g Ni, 3-g MF (M-300) and 12-g NR-latex, and Magnet 2. Figure 11 compares MCF rubber liquid before the application of electric and magnetic fields with that of MCF rubber of the anode-side surface solidified by the DM with a magnetic field. It is well known that NR-latex contains molecules of polyisoprene, as shown in Figure 12. The characteristic peaks are at 1663 and 1710 cm^−1^ (C=C stretching for carbon double bonds of isoprene); 2852, 2915 and 2960 cm^−1^ (CH_2_ stretching vibration of C-CH_2_CH_3_ of isoprene); 3036 cm^−1^ (C-H stretching vibration of isoprene); in addition, at 1000 cm^−1^ (C-C of joint between each isoprene). Comparison of the peak at 3036 cm^−2^ of C-H, which does not contribute to the electrolytic polymerization, with the peaks at 1663 and 1710 cm^−1^ of C=C of electrolytically polymerized MCF rubber, and at 1000 cm^−1^ of C-C are estimated and presented in Table 2. Results indicate the typical characteristic that the quantity of C=C and C-C bonds increase by electrolytic polymerization. 

**Figure 13:**
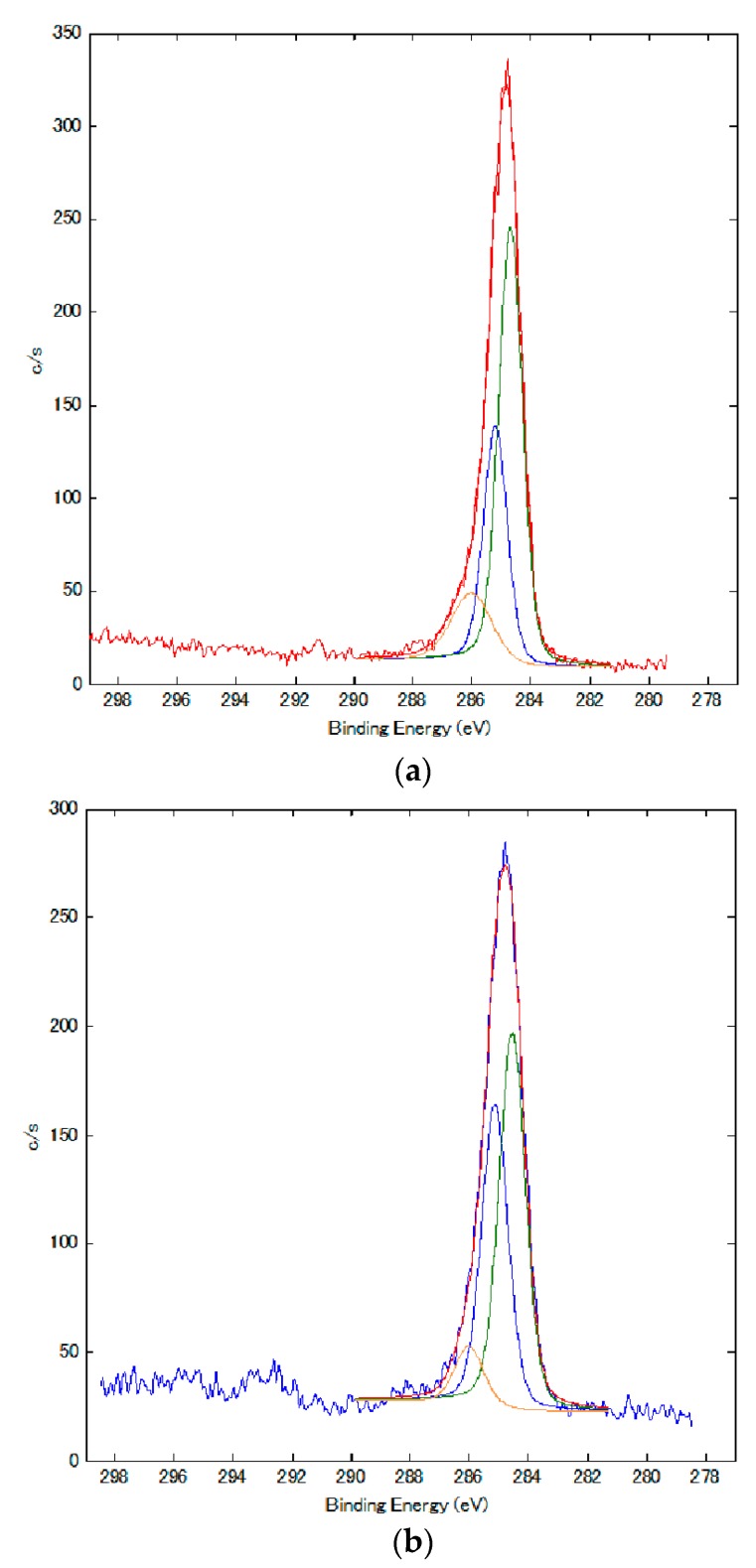
XPS spectra of MCF rubber of anode-side surface: (**a**) electrolytically polymerized MCF rubber; (**b**) MCF rubber solidified by the DM with a magnetic field.

The electrolytically polymerized MCF rubber of the anode-side surface in Figure 11 was analyzed by XPS. Figure 13 presents the results. The MCF rubber was electrolytically polymerized under the same experimental conditions as in Figure 11. Figure 13 shows a comparison with rubber solidified by the DM. 

Comparing polarization of the spectra of C to those of C=C (green line in Figure 13) and C-C (blue and yellow lines), the quantity of C-C blue line in Figure 13b is increased from that in Figure 13a. Therefore, the quantity of C-C bonds increases by electrolytic polymerization. From Figure 11 and Figure 13, it can be deduced that by electrolytic polymerization of the MCF rubber, some polymerization occurs.

The changes in quantity of C=C and C-C bonds is related to the electro-chemical reaction, which in turn is based on the oxidation-reduction reaction. This can be evaluated by [OH^−^] and [H^+^] by measuring the values of redox potential (ORP) and pH. 

It is well known that NR-latex surface particles have a negative charge in their initial condition. When the water in NR-latex reacts as in Equation (1), hydrogen ions H^+^ attract some anionic NR-latex particles, neutralized particles are aggregated, and a part of the NR-latex may be solidified, which does not mean vulcanization. This phenomenon is equivalent to solidification by drying in the air (i.e., the DM):

H_2_O → H^+^ + OH^−^ (1)

**Figure 14:**
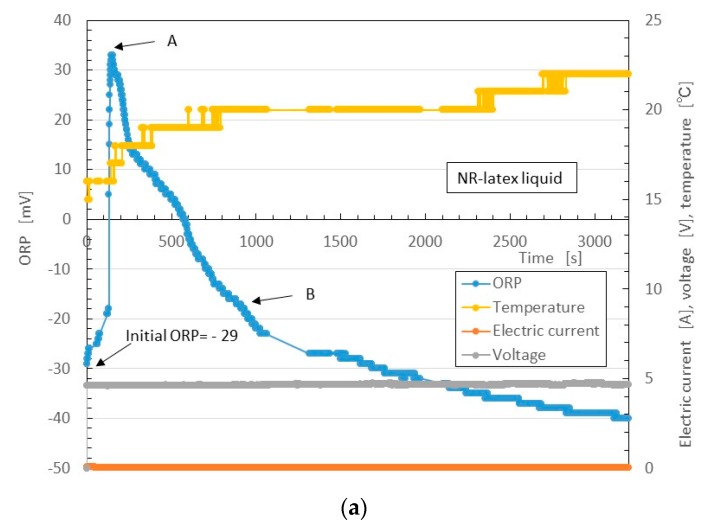

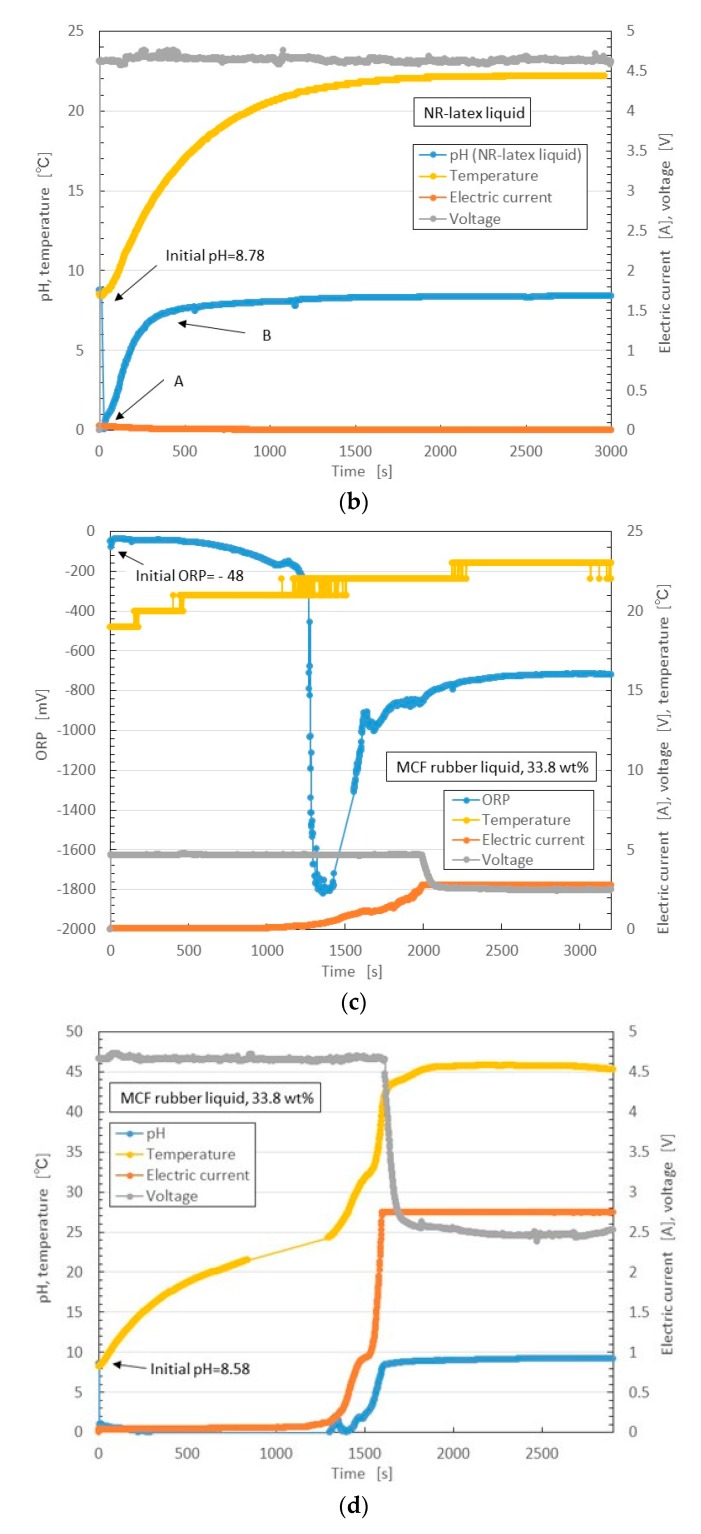
Changes in ORP, pH, temperature, electric current and voltage over time during electrolytic polymerization by the NM: NR-latex liquid in (**a**,**b**); MCF rubber liquid in (**c**,**d**).

Therefore, the quantity of [OH^−^] is greater than that of [H^+^]. As a result, NR-latex is alkaline. This is before the application of the electric field. By electrolytic polymerization, the [OH^−^] and [H^+^], or the ratio of oxidation and reduction, is altered. Thus, by electric-chemical measurement of ORP and pH, we can determine the reaction of the electrolytic polymerization of the MCF rubber based on the physical model shown in Figure 8. We therefore investigated the ORP and pH of the MCF rubber under the application of electric and magnetic fields. The liquid in the MCF rubber consisted of 12 g-Ni powder, 3-g MF (W-40) and 24-g NR-latex; it was poured into a container, as shown in Figure 1. The mass concentration of the MCF rubber liquid was 33.8 wt % of Ni and Fe_3_O_4_ particles. ORP and pH meters were settled between the electrodes immersed in the MCF rubber liquid. Magnet 2 was applied from outside the container. The electric field was held constant at 6 V, and 2.7 A was supplied between the plates. The plates were held apart at a constant thickness using a spacer with a 19-mm electrodes gap, creating a 40 mm × 40 mm square of stainless steel. Figure 14 shows the results. 

First, we consider the NR-latex liquid. As mentioned, in its initial condition, the pH of NR-latex liquid is alkaline as shown in Figure 14b. This state is the same as that of the MCF rubber liquid (see Figure 14d). At the anode, by the application of the electric field, OH^−^ from Equation (1) changes by radial reaction with indicating by as follows:

OH^−^ → OH^・^ + e^−^ (2)

However, the Equation (2) reaction of does not occur at the initial condition, leaving the NR-latex liquid alkaline. 

At the initial condition, the quantity of reductant [red] is larger than that of oxidizer [ox]. Therefore, the ORP of the NR-latex liquid is negative, as shown in the initial ORP in Figure 14a. Equation (2) occurs with the application of the electric field, and [OH−] decreases, becoming smaller than [OH^+^]. Therefore, ORP changes from negative to positive, as shown by A in Figure 14a (hereafter, Reaction A). The pH becomes acidic, as shown by A in Figure 14b. In other words, the oxidation process occurs at the anode by the application of the electric field.

Next, anionic NR-latex particles are attracted to H^+^, and [OH^−^] becomes higher than [H^+^]. Therefore, the pH of the NR-latex liquid changes to alkaline, as shown by B in Figure 14b (hereafter, Reaction B); the [red] becomes larger than [ox]. ORP then changes to negative, as shown by B in Figure 14a.

Next, we consider the MCF rubber liquid. Because it involves NR-latex, the pH and ORP results described can be compared to those of MCF rubber liquid, as shown in Figure 14c,d. These are the reactions at the anode. Meanwhile, at the cathode, the oleic acid undergoes a radial reaction as follows:

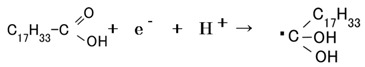
(3)

The possibility of this reaction has already been presented [36,37]. Figure 7 indicated that MF is an important factor in the enhancement of the solidification of MCF rubber. Therefore, we focus on the role of oleic acid in electrolytic polymerization.

In Equation (3) [red] becomes larger than [ox], the ORP of MCF rubber liquid becomes negative, and [OH^−^] becomes higher than [H^+^]. Therefore, the pH of the NR-latex liquid becomes alkaline. At this time, Reactions A and B occur simultaneously at the cathode. Since the data of Figure 14 are between the electrodes, they are measured as a mix of the reactions at the anode and cathode. Therefore they are summarized in the results of ORP and pH shown in Figure 14c,d, respectively.

From the above results, we can construct the process of electrolytic polymerization. As shown in the previous Section 2, enhancement of the solidified MCF rubber with needle- or wire-like shapes by electrolytic polymerization is due to the effect of the magnetic field. It particularly depends on the MF involved in the MCF rubber liquid. The oleic acid coating Fe_3_O_4_ of MF is thought to play a role in the solidification. In addition, as previously shown, C-C bonds increase, and changes in pH and ORP occur by the application of the electric field, confirming electrolytic polymerization of MCF rubber. Based on these results, we hypothesize that the electrolytic polymerization process proceeds as follows. First, at the anode, the anion of the NR-latex particle (Figure 12) is thought to change as follows:

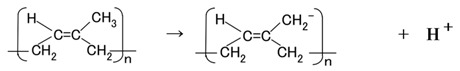
(4)

From Equation (4) we derive Equation (5).

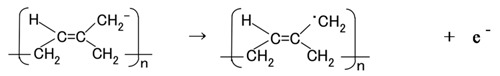
(5)

The possibility of the reaction of Equation (5) has already been presented [36]. The first term on the right side in Equation (5) is rewritten as **^・^**Br for simplicity, and the polyisoprene shown in Figure 12 to RH is rewritten as follows to R.

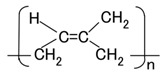
(6)

From the right side in Equation (5) we derive Equation (7) as presented in a previous study [37].

(7) $${}^{・}\mathrm{Br}+\mathrm{RH}\;\to \;\mathrm{Br}\;\dot{R}\;H\;\to \;\mathrm{RBr}+H{}^{・}$$

RBr is created by radical polymerization, as shown below in Equation (8). Therefore, as shown in Figure 11 and Figure 13, C-C bonds increase and MCF rubber liquid is vulcanized as shown by B and G in Figure 8.

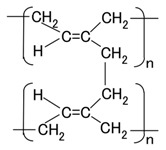
(8)

Here, the following reaction may occur,

H**^・^** + OH**^・^**  →  H_2_O (9)

Next, at the cathode, the radicals of the right side in Equation (3) and **^・^**Br react as follows,

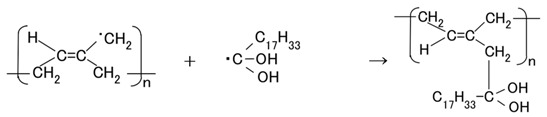
(10)

Therefore the NR-latex and oleic acid are vulcanized as shown by D in Figure 8. We have already demonstrated that the electrolytic polymerization of NR-latex is different at the anode and cathode sides of the MCF rubber surface (Figure 10). Therefore, electrolytic polymerization of the MCF rubber is different at the anode and cathode sides, as shown in Equations (8) and (10).

The vulcanization phenomenon of MCF rubber liquid is similar to that of conductive polymers such as polyacetylene. Equations (2) and (5) describe how an electron occurs and moves through π-conjugation as π-electron, like the carrier of dopant in the C=C bond. As shown by B and G in Figure 8, bonds of C=C and C-C are connected like a network. Therefore, electric conductivity is enhanced and electrical resistivity reaches on the order of 10^−3^ Ω·m, as shown in our previous study [22]. As a reference, polyphenylene-based conductive plastic doped with ASF_5_ is on the order of 10^−2^ Ω·m. On the other hand, ordinary metals such as silver and copper are on the order of 10^−7^ Ω·m. By the NM, we obtained a soft and highly conductive material with high sensitivity that could be applicable to sensors. In addition, because the bonds of C=C and C-C produced by the electrolytic polymerization have large bonding force, it makes the MCF rubber long-term stability of electric conductivity.

## 4. Conclusions

Our aim was to develop a new manufacturing process for NR-latex as a soft rubber embedded with MCF as magnetic filler using the NM. The effect of the magnetic field on the metamorphosis of MCF rubber by electrolytic polymerization and the process of electrolytic polymerization were clarified.

We first clarified how MCF rubber can be enhanced by electrolytic polymerization. The MF in MCF rubber liquid is a very important factor in the enhancement of wire-shaped solidified MCF rubber. The magnetic field distribution is also significant. Magnetic field gradient and strength must be considered in order to obtain large solidified MCF rubber. Specifically, MCF rubber liquid with 21.7 wt % can be solidified at less than about 50-mT magnetic field strength, and enhanced at the position between the maximum magnetic field strength and maximum magnetic field gradient. This enhanced MCF rubber depends on the aggregation of magnetic clusters aligned along the magnetic field line, like needles. We clarified that this enhancement of MCF rubber by electrolytic polymerization is based on radical polymerization, as shown in Equation (3). The production of NR-latex MCF rubber by the NM could be related to production of a highly sensitive sensor.

SEM and XRD images revealed that electrolytically polymerized MCF rubber presents a complicated configuration of intertwined NR-latex, Fe_3_O_4_ and Ni. They also clarified that electrolytic polymerization of NR-latex is different at the anode and cathode sides of the MCF rubber surface. Therefore, electrolytic polymerization of MCF rubber is also different at the anode and cathode sides, as shown in Equations (8) and (10). Raman spectroscopy and XPS showed that the quantity of C=C and C-C bonds increases by electrolytic polymerization.

Meanwhile, the chemical approach revealed that the pH and ORP of MCF rubber liquid is altered by electrolytic polymerization. Based on these results, we were able to propose a hypothetical process for electrolytic polymerization of MCF rubber, as shown in the physical model. We clarified why NR-latex MCF rubber produced by the NM has high sensitivity and long-term conductive stability. Therefore, we showed that the NM may be useful in the development of a high-sensitivity sensor. Because the new production of NM uses small quantity of voltage, electric current and magnetic field strength in the application of electric and magnetic fields, it brings about such an easy implementation that the sensing fields would develop.

## Figures and Tables

**Table 1:** Magnetic field strength of Magnets 1–4.

Location	Magnet 1	Magnet 2	Magnet 3	Magnet 4
at b or c in Figure 1 (mT)	34.5	48.3	80.1	107
at a in Figure 1 (mT)	22.0	28.1	43.4	55.3

**Table 2:** Comparison of spectra from Figure 11.

Location	at 1663 and 1710 cm^−1^ of C=C	at 1000 cm^−1^ of C-C
Electrolytically polymerized MCF rubber	55.88	12.94
MCF rubber liquid before electrolytic polymerization	13.18	3.21
MCF rubber solidified by Drying Method with magnetic field	13.73	3.36
